# The distinct and potentially conflicting effects of tDCS and tRNS on brain connectivity, cortical inhibition, and visuospatial memory

**DOI:** 10.3389/fnhum.2024.1415904

**Published:** 2024-05-30

**Authors:** Pei-Jung Wu, Chih-Hsu Huang, Shuenn-Yuh Lee, Alice Y. W. Chang, Wen-Chi Wang, Chou-Ching K. Lin

**Affiliations:** ^1^Department of Neurology, College of Medicine, National Cheng Kung University, Tainan, Taiwan; ^2^Department of Electrical Engineering, National Cheng Kung University, Tainan, Taiwan; ^3^Department of Physiology, College of Medicine, National Cheng Kung University, Tainan, Taiwan; ^4^National Cheng Kung University Hospital, College of Medicine, National Cheng Kung University, Tainan, Taiwan

**Keywords:** fMRI, tDCS, tRNS, temporal cortex, brain connectivity, cortical inhibition, visuospatial memory

## Abstract

Noninvasive brain stimulation (NIBS) techniques, including transcranial direct current stimulation (tDCS) and transcranial random noise stimulation (tRNS), are emerging as promising tools for enhancing cognitive functions by modulating brain activity and enhancing cognitive functions. Despite their potential, the specific and combined effects of tDCS and tRNS on brain functions, especially regarding functional connectivity, cortical inhibition, and memory performance, are not well-understood. This study aims to explore the distinct and combined impacts of tDCS and tRNS on these neural and cognitive parameters. Using a within-subject design, ten participants underwent four stimulation conditions: sham, tDCS, tRNS, and combined tDCS + tRNS. We assessed the impact on resting-state functional connectivity, cortical inhibition via Cortical Silent Period (CSP), and visuospatial memory performance using the Corsi Block-tapping Test (CBT). Our results indicate that while tDCS appears to induce brain lateralization, tRNS has more generalized and dispersive effects. Interestingly, the combined application of tDCS and tRNS did not amplify these effects but rather suggested a non-synergistic interaction, possibly due to divergent mechanistic pathways, as observed across fMRI, CSP, and CBT measures. These findings illuminate the complex interplay between tDCS and tRNS, highlighting their non-additive effects when used concurrently and underscoring the necessity for further research to optimize their application for cognitive enhancement.

## 1 Introduction

Noninvasive brain stimulation (NIBS) techniques, such as transcranial direct current stimulation (tDCS) and transcranial random noise stimulation (tRNS), have gained significant research attention for their potential in augmenting cognitive functions and discovering mechanisms and treatments in neurological diseases (Marshall et al., 2005; Fregni et al., 2006; Liebetanz et al., 2006; Brunoni et al., 2019). These techniques modulate brain functions by influencing neuronal excitability and regulating the activity of specific cerebral regions, consequently impacting the brain’s functional networks (Esmaeilpour et al., 2020).

tDCS utilizes low-intensity direct current to influence neuronal activity. Its precise mechanism is still under investigation (Bestmann and Walsh, 2017). Generally, it is thought that tDCS may affect the resting membrane potential of neurons (Radman et al., 2009; Kronberg et al., 2017), thereby affecting neuronal excitability and synaptic plasticity (Marquez-Ruiz et al., 2012; Stagg et al., 2018). Additionally, the polarity of the electrodes used in tDCS (anodal or cathodal) represents the direction of these effects, with anodal stimulation presumably leading to an increase in excitability and cathodal stimulation resulting in decreased excitability (Nitsche et al., 2005; Li et al., 2019).

tRNS, another stimulation protocol similar to tDCS, uses alternating current at random frequencies, as opposed to a constant current (van der Groen et al., 2022). The hypothesized mechanism of tRNS is to induce stochastic resonance by enhancing the level of neuronal noise (van der Groen and Wenderoth, 2016). Both tDCS and tRNS havebeen associated with increased cortical excitability and improving cognitive performance, although the underlying mechanisms are thought to differ due to its current nature (Nitsche et al., 2008; Terney et al., 2008; Moliadze et al., 2010).

The integration of tDCS with tRNS is commonly referred to as tRNS with a DC bias. While the underlying mechanisms are still unknown, several potential mechanisms, such as synergistic modulation of cortical excitability, enhanced synaptic plasticity, and augmented stochastic resonance, have been proposed (Ho et al., 2015; Peña et al., 2023). This combined paradigm is relatively novel, as existing NIBS research primarily focuses on either tDCS or tRNS alone. Few studies have explored their interaction, and to our best knowledge, none have yet investigated their synergistic effects using neuroimaging approaches.

The objective of this research was to investigate the distinct and combined effects of tDCS and tRNS when applied to the temporal cortex, a critical region for memory functions. Employing a within-subject design, our investigation extended to evaluating the impacts of these neuromodulatory techniques on resting-state functional connectivity among key cerebral regions. The rationale for focusing on the resting-state condition lies in its direct reflection of the brain’s intrinsic response to electrical stimulation. This approach bypassed the variables associated with task performance enhancement and was instrumental in clarifying the broader network functionality and activity within the brain (Mondino et al., 2019). In addition, our study recorded the effects of these stimulation protocols on cortical inhibition and visuospatial memory performance, thus offering a holistic view of their modulatory capabilities.

## 2 Materials and methods

### 2.1 Participants

Ten male participants (ages 21–25 years) from the student population were recruited on campus. All participants reported to be healthy with no history of neurological disorders or contraindication to transcutaneous electric stimulation, transcranial magnetic stimulation (TMS) or magnetic resonance imaging (MRI). This study was performed in accordance with the ethical guidelines and was approved by the Institutional Review Board of National Cheng Kung Hospital, Tainan, Taiwan. Informed consent was obtained from all participants prior to the experiments.

### 2.2 Designs

**Figure 1:**
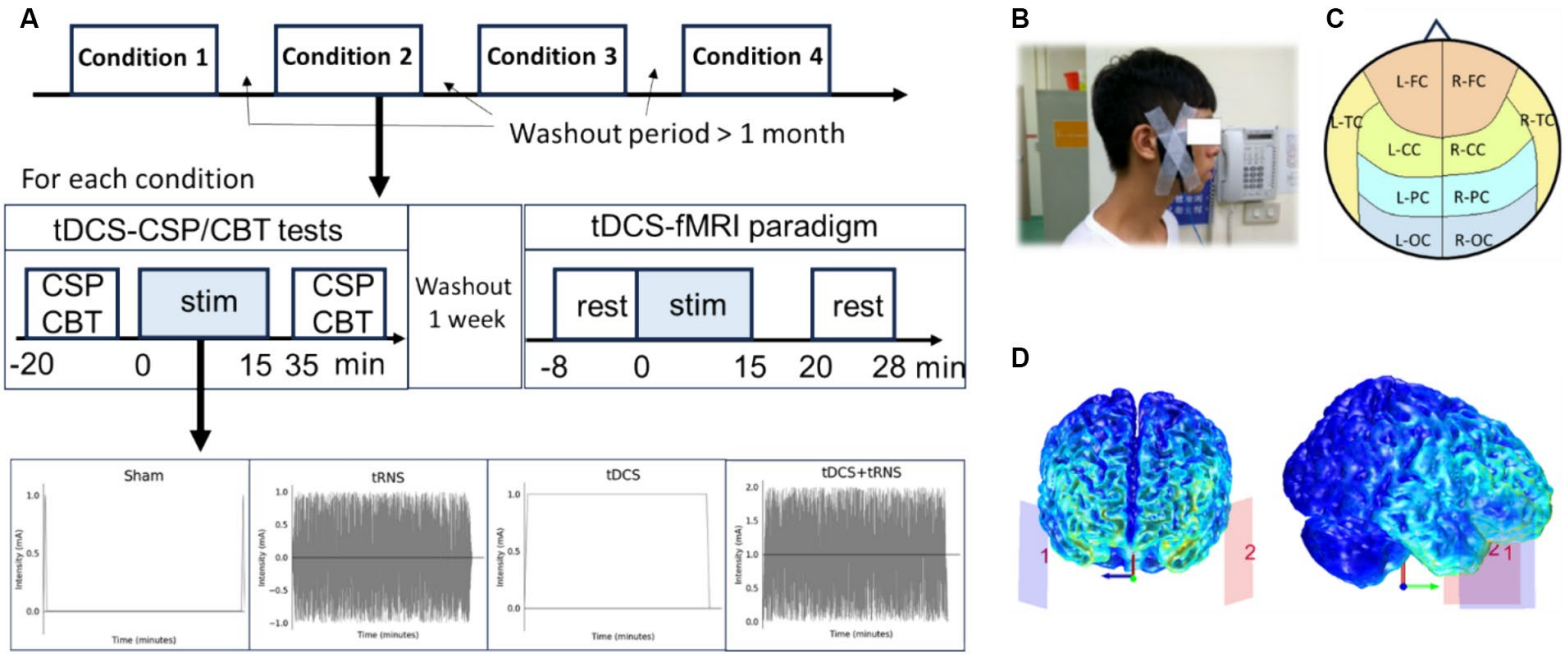
Overview of the study design investigating the effects and interactions of tDCS and tRNS. **(A)** Illustration of the placebo-controlled, within-subject experimental design for four conditions: sham, tDCS, tRNS, and combined tDCS + tRNS, with a minimum one-month interval between each condition. **(B)** Electrode placement on the temple region, with the anodal on the left side. **(C)** Representation of defined cerebral cortex regions, including L-FC, left frontal cortex; R-FC, right frontal cortex; L-CC, left central cortex; R-FC, right central cortex; L-TC, left temporal cortex; R-TC, right temporal cortex; L-PC, left parietal cortex; R-PC, right parietal cortex; L-OC, left occipital cortex; R-OC, right occipital cortex. **(D)** Computational simulation of electric field distribution during temporal transcranial direct current stimulation (tDCS).

A single-blind, placebo-controlled, within-subject design, deploying a randomized controlled trial framework was conducted to understand the effects of two distinct NIBS modalities: tDCS and tRNS. Ten participants underwent four different intervention conditions in a randomized order: sham, tDCS, tRNS, and tDCS + tRNS, each for a duration of 15 min. The study was structured into separate sessions for functional magnetic resonance imaging (fMRI) and for the Cortical Silent Period (CSP)/Corsi Block-Tapping (CBT) tests. Between these sessions, a one-week washout period was incorporated to reduce potential carryover effects. The fMRI sessions were designed to assess the immediate impact of stimulation on brain connectivity, whereas the CSP and CBT tests in different sessions evaluated cortical inhibition and cognitive memory performance, respectively. Additionally, a more extended washout period of over 1 month was strategically placed between different stimulation conditions to further ensure data integrity (Figure 1A).

### 2.3 Stimulation protocol

NIBS was delivered using an MR-compatible, battery-driven stimulator (neuroConn, Germany). A pair of 5 × 7 cm^2^ rubber electrodes, coated with conductive gel, ensured that contact impedance remained below 50 kΩ. For both tDCS and tRNS modalities, electrodes were positioned to target the temporal cortex (Figure 1B), with electrode placement on either side of the temple area, equivalent to the EEG locations FT9 for the anode and FT10 for the cathode. An electric field simulation obtained using the COMETS Toolbox (version 2.0) (Lee et al., 2017) confirmed that this montage was appropriate to entrain neural activity in the temporal cortex (Figure 1D). According to the design, 1 mA tDCS, 2 mA (peak-to-peak amplitude) tRNS with frequencies ranging from 100 to 640 Hz, or combined these two conditions, was applied for 15 min with a 15-s ramp-up/down phase. For sham, only ramp-up/down phases with no stimulation were applied.

### 2.4 Cortical silent period (CSP) testing

Cortical silent period was assessed using single-pulse transcranial magnetic stimulation (TMS) over the left motor cortex. The procedure was implemented using a MagStim 200 stimulator (Magstim Co. Ltd., United Kingdom). Participants were required to maintain a 40% maximum force grip with their right hand while the EMG activity of the thenar muscle was recorded. The resting motor threshold was established before measurements, and the TMS pulse was set to 120% of this threshold. EMG signals were recorded at a sampling rate of 2 kHz using an EMG100C amplifier (Biopac System, Inc., United States). The CSP duration, indicative of GABAb-mediated cortical inhibition, was estimated by an automated algorithm (Julkunen et al., 2013).

Despite the primary stimulation site in our study was the temporal cortex, the broad electric field distribution inherent in transcranial electrical stimulation techniques like tDCS and tRNS can affect regions beyond the targeted area, including the motor cortex. This influence of electrical currents extends to areas susceptible to neuromodulatory effects, even those not directly under the electrodes. This methodological choice is supported by analogous approaches in epilepsy research, where changes in motor threshold and MEP are used to assess the impact of treatments across the brain, highlighting the systemic effects of therapeutic interventions (Badawy et al., 2013). Using CSP allows us to infer the effects of stimulation on cortical inhibition broadly, given current limitations in directly measuring such effects in non-motor regions like the temporal cortex. This approach underscores the exploratory nature of our study, aiming to broaden the understanding of how different forms of brain stimulation interact and affect neural function across the cortex.

### 2.5 Corsi block-tapping (CBT) test

The CBT test consisted of encoding and recalling phases, designed to assess memory and sequencing capabilities. The initial span was set to 2 blocks, increasing by one after two successful trials and continuing until the participant failed two consecutive trials at the same level. During the encoding phase, participants memorized the sequence of color-changing blocks on the screen. In the recalling phase, they were required to select the blocks in the same or reverse order. The CBT performance was quantified as the sum of spanned blocks correctly recalled across all difficulty levels. We also recorded the reaction time. The interference conditions aimed to challenge the working memory further during the encoding phase. The completed blocks, accuracies, and reaction times were recorded for analysis.

### 2.6 MRI data acquisition

MRI scans were performed on a 3.0T GE 750 scanner (GE Healthcare Systems) equipped with an 8-channel head coil to ensure high-resolution data capture. Structural brain images were captured using a T1-weighted inversion prepared 3D spoiled gradient echo (IR-SPGR) sequence. This sequence provided detailed anatomical information with a field of view (FOV) of 224 × 224 mm^2^, a time of repetition (TR) of 7.7 ms, a time of echo (TE) of 2.9 ms, and an image matrix of 224 × 224, resulting in 170 sagittal slices with each 1 mm thick. For functional imaging, a T2-weighted echo-planar imaging (EPI) sequence was implemented, optimized for resting-state fMRI, with a TR of 2000 ms, TE of 30 ms, and a flip angle set at 90 degrees. This functional protocol acquired 40 axial slices with an in-plane resolution of 64 × 64 and a slice thickness of 3 mm, generating 240 volumes per subject.

### 2.7 Image preprocessing

MRI data preprocessing was performed using SPM12 in MATLAB R2022b (MathWorks, Inc., United States). We first applied slice time correction, followed by realignment protocols to mitigate effects of participant motion. A 6 mm FWHM Gaussian kernel was employed for spatial smoothing, enhancing signal-to-noise ratios and normalizing for inter-individual anatomical variations. Co-registration was done to align functional EPI with structural scans, while bias-field correction adjusted for magnetic field inconsistencies, thereby refining signal quality for subsequent connectivity analyses. In addition, functional data were denoised using a standard denoising pipeline built in the CONN toolbox (Whitfield-Gabrieli and Nieto-Castanon, 2012).

### 2.8 Connectivity analyses

Regional and network-based analyses were performed using the CONN toolbox in MATLAB (Whitfield-Gabrieli and Nieto-Castanon, 2012), with the Harvard-Oxford atlas employed for defining Regions of Interest (ROIs). In the regional analysis, 10 brain regions were delineated based on anatomical location: left and right frontal (L-FC, R-FC), central (L-CC, R-CC), temporal (L-TC, R-TC), parietal (L-PC, R-PC), and occipital cortices (L-OC, R-OC) (see Figure 1C and Supplementary Table S1). We computed a 10×10 correlation difference matrix by subtracting the pre-stimulation correlation matrix from the post-stimulation one to assess the global effects of electrical stimulation on connectivity. Further, we evaluated lateralization and dispersion by assigning scores to each brain region based on *p*-values derived from two-way repeated measures ANOVA: regions with *p* < 0.1 received three points, regions with 0.1 < *p* < 0.2 received two points, and regions with 0.2 < *p* < 0.3 received one point. The dispersion was gauged by the cumulative scores across regions. Lateralization was determined by subtracting the cumulative score of the right hemisphere from that of the left hemisphere.

Network-based analysis was conducted on seven large-scale networks using the first 32 regions in the Harvard-Oxford atlas, including the default mode, sensorimotor, visual, salience, dorsal attention, frontoparietal, and language networks (Supplementary Table S1). For each network, nodes’ connectivity values were averaged, yielding a 7 × 7 correlation difference matrix that offered insights into network-specific connectivity changes following stimulation.

### 2.9 Statistical analysis

We employed a 2 × 2 (tDCS x tRNS) two-way repeated measures ANOVA for analyzing fMRI and CBT data. However, for the CSP results, due to poor quality in two specific data sessions from two different subjects, we resorted to a standard two-way ANOVA, as the incomplete data precluded the use of a repeated measures approach. We then did a post-hoc comparison between sham and tDCS/tRNS for the conditions that have strong interaction (*p* < 0.1) from ANOVA results.

We further analyzed the fMRI results from the statistical analysis for investigating the relationship between different brain regions. To explore the extent of alterations induced by stimulation, we examined the observed changes based on *p*-values. Confidence levels were set with thresholds at *p* < 0.1 for high confidence, 0.1 < *p* < 0.2 for moderate confidence, and 0.2 < *p* < 0.3 for lower confidence.

Based on the confidence levels, functional connectivity brain maps can be visualized. Weights were assigned to each brain region pair according to their p-values, which were then used to calculate lateralization scores for both the left and right hemispheres. Furthermore, large-scale resting-state brain networks were analyzed using the same statistical model. By applying this two-way repeated measures ANOVA, we could assess the broader network-level effects of tDCS and tRNS.

## 3 Results

### 3.1 Impact of temporal tRNS and tDCS on resting state functional connectivity

**Figure 2:**
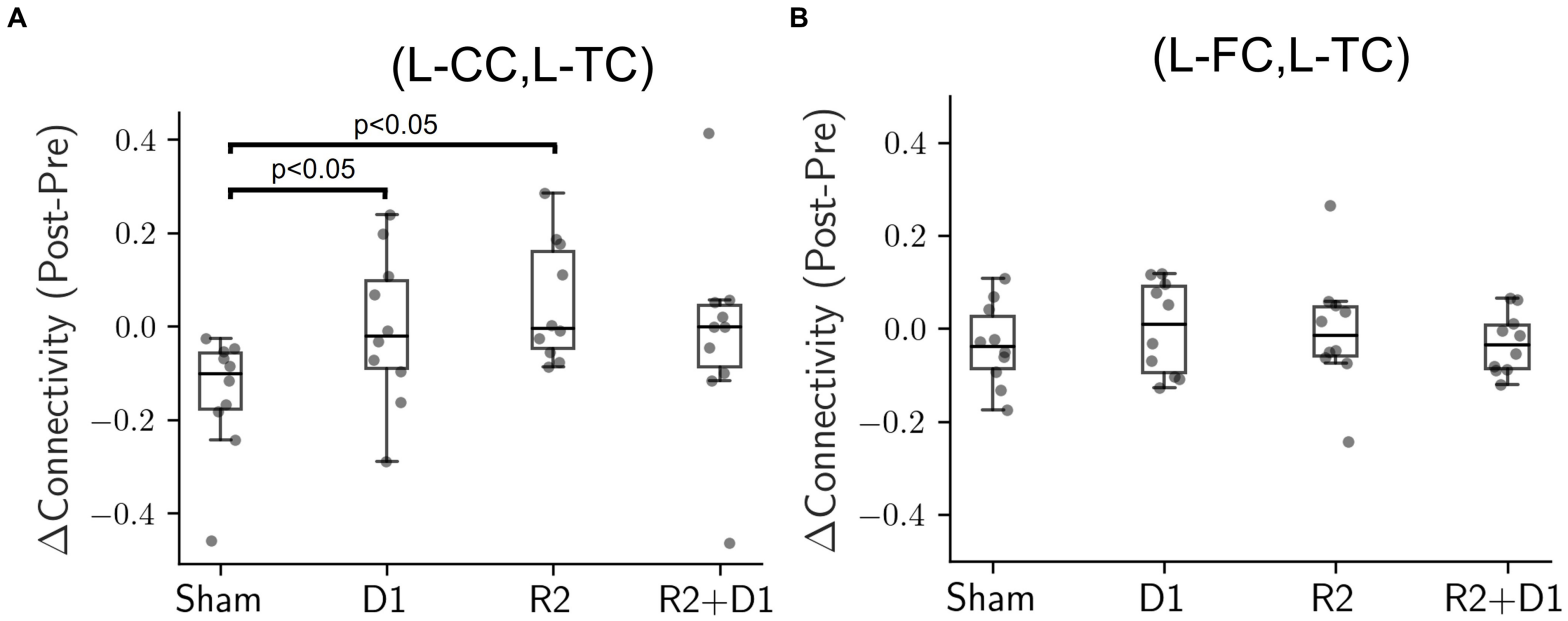
Comparison of connectivity changes under different stimulation conditions. **(A)** Box plot illustrating the connectivity changes between the left central cortex (L-CC) and the left temporal cortex (L-TC), identified as D1 for tDCS and R2 for tRNS. Exploratory paired-t tests, compared to the sham condition, reveal statistically significant changes for tDCS (D1, *p* = 0.047) and tRNS (R2, *p* = 0.014) interventions. However, these initial significant effects dissipated when analyzed using a two-way repeated measures ANOVA. **(B)** Box plot showing connectivity changes between the left frontal cortex (L-FC) and the left temporal cortex (L-TC). Neither the exploratory paired-t tests nor the two-way repeated measures ANOVA showed statistically significant differences in any condition when compared to sham.

In order to investigate the effects of tDCS, tRNS and their interaction, we first employed data visualization and initial exploratory analysis. Using paired-t test on the change of connectivity to see the data at first glance, we found there was a statistically significant level in the change of connectivity between the left central cortex (L-CC) and the left temporal cortex (L-TC) after tDCS, labeled as “D1” in our analysis, *t* (9) = −2.294, *p* = 0.047, and tRNS, labeled as “R2,” *t* (9) = −3.024, *p* = 0.014, interventions (Figure 2A). These preliminary findings provide a hint that the functional connectivity in some brain regions might be affected by either modality.

However, when the analysis was extended to include all four stimulation conditions (sham, D1, R2, and D1 + R2) using a two-way repeated measures ANOVA, the previously significant effects were no longer present. The result suggests that the impact of tDCS and tRNS on brain connectivity might be more complex and nuanced than originally presumed. Particularly, the interaction effects between tDCS and tRNS approached marginal significance [*F* (1,9) = 4.564, *p* = 0.061, $\eta_{p}^{2}$ = 0.102] indicating a potential interplay between the two modalities that did not quite reach conventional levels of statistical significance. In addition, Figure 2B shows that even neighboring brain regions could exhibit varying responses to the same electrical stimulation, indicating that the effects were not uniformly distributed across the cortex. Despite the absence of statistical significance, our findings suggest a spatial pattern in functional connectivity to the effects of tDCS and tRNS.

**Figure 3:**
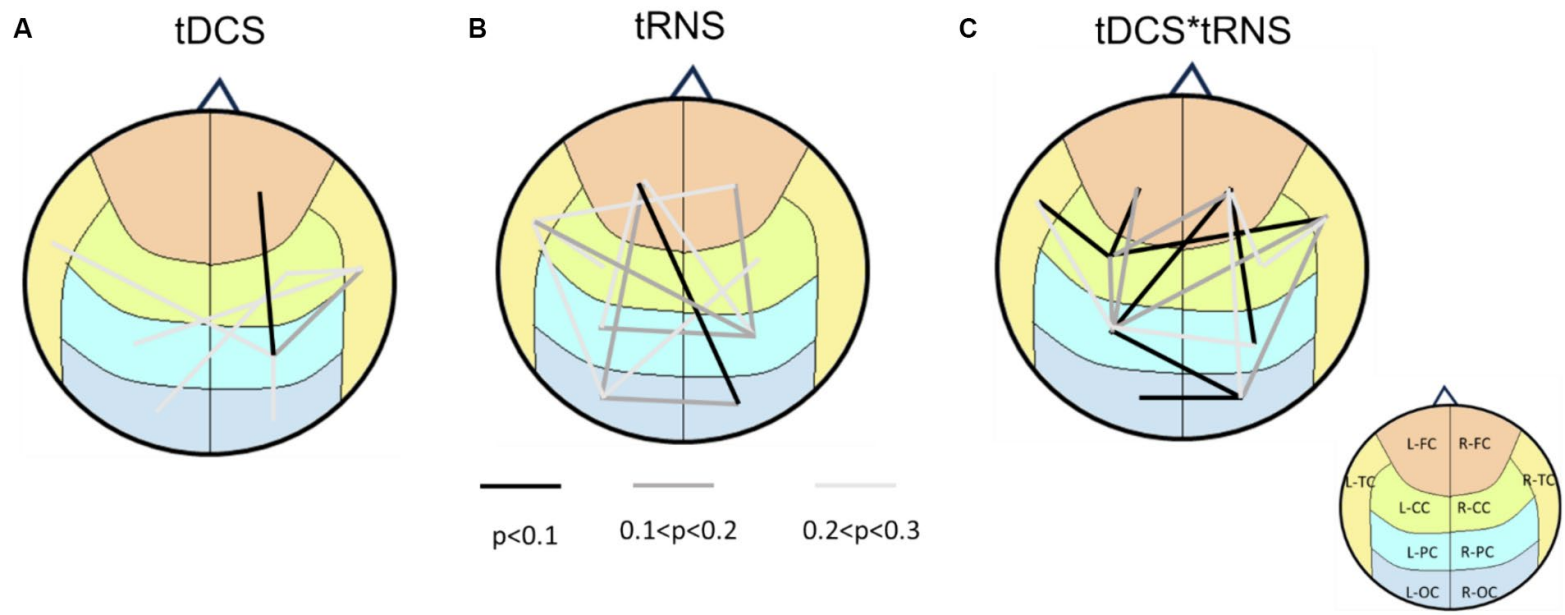
Brain connectivity maps based on *p*-values from two-way repeated measures ANOVA. **(A)** the main effects of tDCS on brain connectivity, indicating right hemisphere lateralization. **(B)** The main effects of tRNS, revealing a more dispersed impact that is slightly right-lateralized. **(C)** The interaction effects between tDCS and tRNS, highlighting that 7 regions exhibited changes with a high degree of confidence (*p* < 0.1). Lines connecting different brain regions indicate the strength of connectivity changes, with color of the lines representing statistical significance levels (refer to the legend for specific *p*-values). The underlying brain regions are demarcated on the map, color-coded according to (Figure 1C) for identification.

To further visualize these effects, we created a brain-wide map that reveals the change induced by stimulation. Based on various *p*-value thresholds as an exploratory approach, these maps revealed that tDCS influenced the right hemisphere more than the left one (Figure 3). In contrast, the impact of tRNS was more dispersed and slightly left-lateralized. The most interesting observations came from the interaction effects, where a significant proportion of brain region pairs (7 pairs) exhibited changes with a high degree of confidence (90%). These changes were not confined to any specific region, indicating a widespread influence of combined stimulation.

### 3.2 Dispersion and lateralization of brain connectivity following tDCS and tRNS stimulation

**Figure 4:**
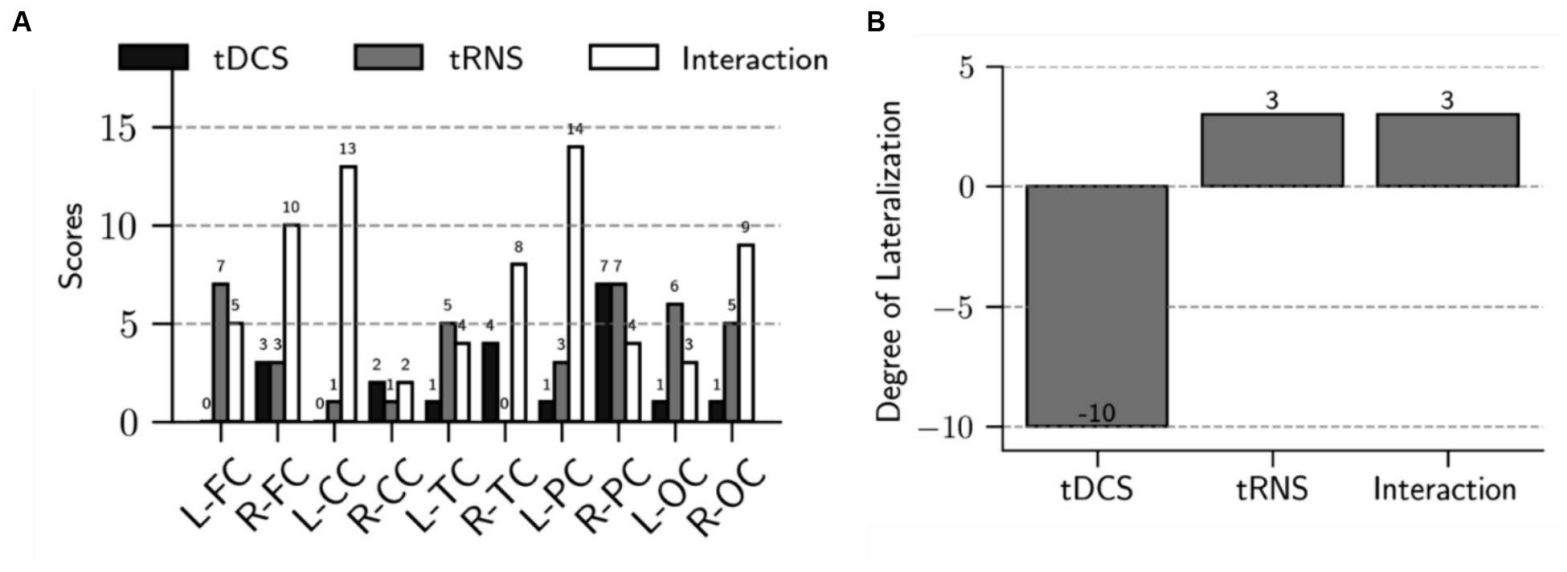
Analysis of dispersion and lateralization of brain connectivity after intervention. **(A)** The bar chart shows the scores for each brain region, highlighting the main effects of tDCS, tRNS, and their interaction. Specifically, it shows a total score of 20 for the tDCS effect, 38 for the tRNS effect, and 72 for the interaction effect. These scores reflect the extent of dispersion across the brain regions following each stimulation condition. **(B)** The bar chart demonstrates the degree of lateralization for the main effects of tDCS, tRNS, and their interaction. Lateralization is calculated by subtracting the right hemisphere’s score from the left hemisphere’s score. The main effect of tDCS is characterized by right hemisphere lateralization, whereas the main effect of tRNS and the interaction between tDCS and tRNS exhibit a slight left hemisphere lateralization.

Our findings demonstrate distinct influences of tDCS and tRNS on brain connectivity. We assessed the dispersion of effects across different brain regions following electrical stimulation. As shown in Figure 4A, after tDCS stimulation, only two brain regions had scores exceeding three, suggesting limited dispersion. In contrast, the tRNS and combined stimulation conditions had a more extensive impact; tRNS influenced five regions with scores above three, and the combined condition affected eight regions. Notably, these affected regions constitute 80% of the total brain regions examined, highlighting a broader range of influence in the combined condition scenarios.

Moreover, Figure 4B shows that tDCS stimulation resulted in a pronounced lateralization towards the right hemisphere, with the lateralization score being 14 points higher than that of the left hemisphere. Conversely, both tRNS and the tDCS + tRNS condition exhibited a preference for left hemisphere lateralization, with a 6-point higher score than the right hemisphere.

### 3.3 Non-synergetic effects of combined tRNS and tDCS

**Figure 5:**
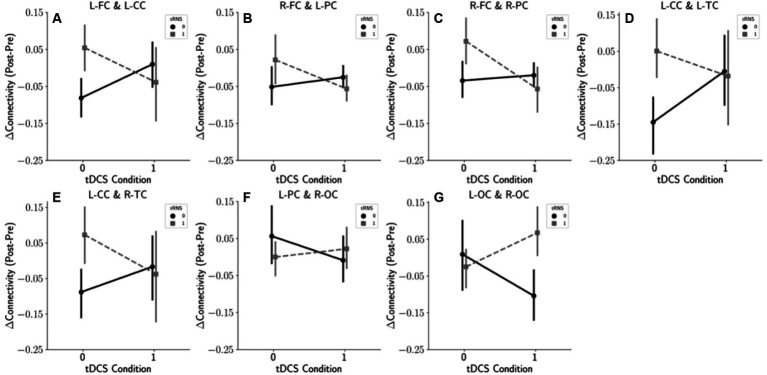
Interaction effects of tDCS and tRNS on the connectivity changes. This figure illustrates the interaction effects based on the results of a two-way repeated measures ANOVA. Panels **(A–G)** display selected brain region pairs where *p*-values fell below the 0.1 threshold, indicating notable interaction effects between tDCS and tRNS. **(A)** Interaction effect between the left frontal cortex (L-FC) and the left central cortex (L-CC). **(B)** Interaction between the right frontal cortex (R-FC) and the left parietal cortex (L-PC). **(C)** Interaction between the right frontal cortex (R-FC) and the right parietal cortex (R-PC). **(D)** Interaction between the left central cortex (L-CC) and the left temporal cortex (L-TC). **(E)** Interaction between the left central cortex (L-CC) and the right temporal cortex (R-TC). **(F)** Interaction between the left parietal cortex (L-PC) and the right occipital cortex (R-OC). **(G)** Interaction between the left occipital cortex (L-OC) and the right occipital cortex (R-OC).

Our statistical analysis suggested a potential interaction between tRNS and tDCS when applied simultaneously on the temporal cortex. To further explore the interplay of these modalities, we employed interaction plots, selectively highlighting brain region pairs with *p*-values under 0.1 (Figure 5A). These plots illustrate that in the majority of anterior brain regions, tRNS outperformed tDCS in modifying connectivity when each was used separately. Intriguingly, the addition of tDCS to tRNS appeared to diminish the effects. On the other hand, for pairs in the posterior regions, an inverse relationship was observed, suggesting a complex and region-specific dynamic between these stimulation methods.

### 3.4 Influence of temporal tRNS and tDCS on resting state large scale brain networks

Using a two-way repeated measures ANOVA, we assessed the impacts and potential interactions of tDCS and tRNS on 7 large-scale brain networks. As shown in Table 1, tDCS demonstrated a tendency to affect the visual network, whereas tRNS exhibited a mild influence on both the default mode network and the frontoparietal network; however, these effects did not reach statistical significance. Contrary to the outcomes of the regional analyses, when tDCS and tRNS were administered concurrently, no particular interaction effects on large-scale brain networks were detected, indicating that the combined neuromodulatory approach does not synergistically alter network-level connectivity.

**Table 1:** The *F*-values and *p*-values from two-way repeated measures ANOVA with factors of tDCS and tRNS.

Network	tDCS *F*-value	tDCS *p*-value	tRNS *F*-value	tRNS *p*-value	tDCS:tRNS *F*-value	tDCS:tRNS *p*-value
Default Mode (DMN)	1.6552	0.2304	4.0815	0.0741	0.1668	0.6925
SensoriMotor (SMN)	0.1868	0.6758	0.8059	0.3927	0.3606	0.5630
Visual (VN)	3.3406	0.1009	1.0357	0.3354	0.2764	0.6118
Salience (SN)	0.0516	0.8254	0.1876	0.6751	0.3087	0.5920
Dorsal Attention (DAN)	0.3719	0.5570	0.6719	0.4335	0.9472	0.3559
FrontoParietal (FPN)	0.1278	0.7290	2.8627	0.1249	0.0110	0.9187
Language (LN)	0.2169	0.6524	1.6834	0.2267	0.9432	0.3568

### 3.5 Modulation of cortical inhibition by temporal tRNS and tDCS

**Figure 6:**
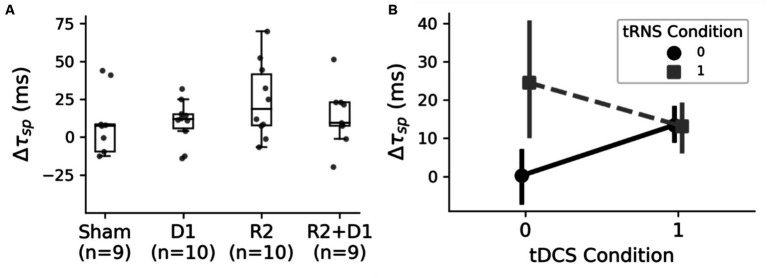
Analysis of the CSP changes under different stimulation conditions. **(A)** The box plot presents the variations in the Cortical Silent Period (CSP) across different stimulation settings. Following the exclusion of outliers, a two-way ANOVA was conducted, revealing a statistically significant main effect of tRNS, as indicated by *F* (1,26) = 4.24 and *p* = 0.049. Additionally, a marginal interaction was observed between tDCS (D1) and tRNS (R2), with *F* (1,26) = 4.14 and *p* = 0.052. **(B)** The interaction effects of tDCS and tRNS on CSP alterations. Notably, tRNS stimulation resulted in a prolonged CSP, whereas the addition of tRNS to tDCS did not produce a cumulative effect, indicating a nuanced interplay between these stimulation methods.

Our study expanded beyond examining functional connectivity to investigate cortical inhibition post-stimulation using the Cortical Silent Period (CSP) metric, as illustrated in Figure 6A. During our analysis, data from two sessions were excluded due to poor signal quality. This exclusion necessitated a modification in our statistical approach; instead of employing a two-way repeated measures ANOVA, we opted for a two-way ANOVA as a viable alternative.

The initial analysis using this two-way ANOVA did not indicate significant changes. However, upon refining our analysis to exclude outliers, we observed a statistically significant main effect of tRNS, with *F* (1,26) = 4.24 and *p* = 0.049. Additionally, there was a notable interaction between tDCS and tRNS, indicated by *F* (1,26) = 4.14 and *p* = 0.052. The interaction plot, as shown in Figure 6B, suggests that the inhibitory effect induced by tRNS was more pronounced. Interestingly, the combined application of tRNS and tDCS yielded effects similar to those observed with tDCS alone. We then did paired-t tests with the original data, and we found that there was a marginal significance between sham and tRNS conditions [*t* (9) = 2.059, *p* = 0.078].

### 3.6 Evaluating the impact of temporal tRNS and tDCS on cognitive memory performance

**Figure 7:**
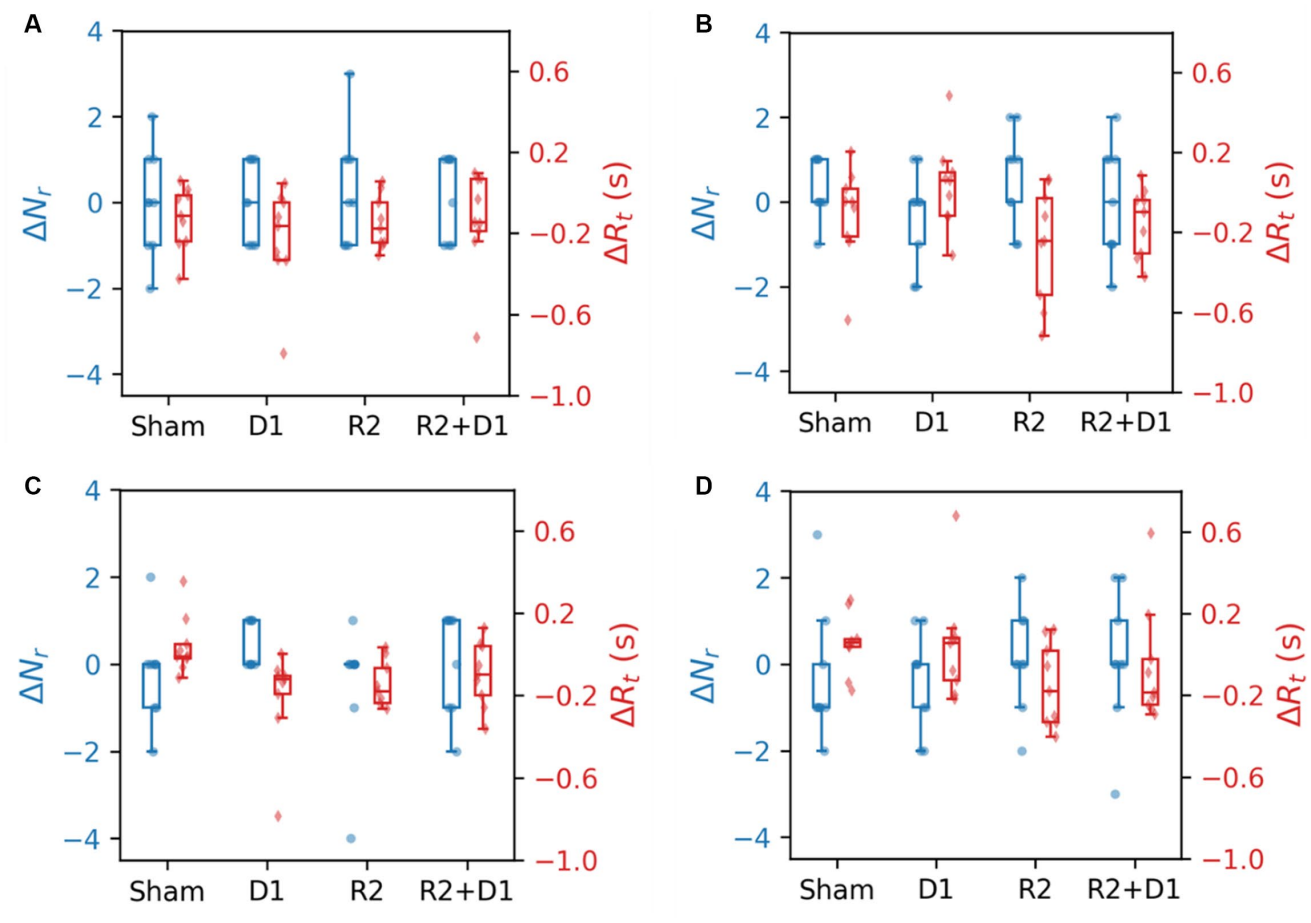
Comparison of CBT performance and reaction time under different stimulation conditions. **(A)** The blocks were selected in the same order. **(B)** The blocks were selected in the reverse order. **(C)** The blocks were selected in the same order with interference. **(D)** The blocks were selected in the reverse order with interference.

Figure 7 displays the CBT performance under various task conditions, measuring the number of blocks completed and reaction times. The dataset represents nine participants due to the absence of one subject’s data. Analysis conducted through a two-way repeated measures ANOVA revealed a statistically significant main effect of tDCS on CBT performance in the Forward Interference (FI) condition [*F* (1,8) = 14.235, *p* = 0.005, $\eta_{p}^{2}$ = 0.080], indicating an improvement in performance without altering reaction times. No statistically significant interaction effect on performance was detected; however, reaction times were significantly affected by the interaction [*F* (1,8) = 5.839, *p* = 0.042, $\eta_{p}^{2}$ = 0.128].

In the backward (B) task condition, stimulation did not significantly differentiate performance outcomes, yet tRNS notably reduced reaction times [*F* (1,8) = 22.659, *p* = 0.001, $\eta_{p}^{2}$ = 0.124]. This effect was not replicated with the tDCS or the tDCS + tRNS combination. A similar trend was observed under backward interference (BI) conditions, where the main effect of tRNS approached marginal statistical significance [*F* (1,8) = 3.781, *p* = 0.088, $\eta_{p}^{2}$ = 0.107]. We then did a paired-t test for reaction time, we found that there is a statistically significant effect between sham and tDCS [*t* (9) = −2.687, *p* = 0.027] and a marginal significance between sham and tRNS [*t* (9) = −2.059, *p* = 0.075].

## 4 Discussion

The aim of this study was to investigate the effects and interactions of tDCS and tRNS over the temporal cortex, revealing a wide range of impacts on functional connectivity. Our exploratory statistical analysis helped identify relationships between brain regions under different protocols of stimulation, providing valuable insights for further research.

During stimulation, the electric field distribution for traditional electrode montages is widespread, indicating a broad area of influence for electrical stimulation (Dasilva et al., 2012). It has been observed that activation induced by electrical stimulation is not confined to the region of stimulation (Baudewig et al., 2001; Stagg et al., 2009), suggesting a broader range of effects due to extensive field distribution or factors related to functional connectivity (Ghobadi-Azbari et al., 2021; Bouchard et al., 2023). Traditional region of interest (ROI) analysis, which often selects small regions as seeds assuming independence among them, might overlook potential covariates or interactions within local areas (Bouchard et al., 2023). Given these considerations, our study employed an analysis method that averages data over larger brain regions—each composed of multiple ROIs. While this approach inherently reduces the sensitivity of detecting subtle changes, making statistical significance more challenging to achieve, it decreases the risk of false positives and enhances our ability to detect genuine, widespread neural effects. Accordingly, we adopted a *p*-value threshold of 0.1 to accommodate the reduced effect size and increased noise reduction, balancing the need for statistical rigor with the exploratory nature of our study. This methodological choice allows for a more comprehensive understanding of the complex and extensive impacts of electrical stimulation on functional connectivity.

We reported that tDCS primarily induced right-sided lateralization, while tRNS led to a slight leftward shift, affecting a broader range of brain areas. This finding suggests a potential mechanistic conflict between tDCS and tRNS, possibly due to their distinct mechanisms. A closer examination reveals that in anterior brain regions, tRNS alone slightly surpassed tDCS in effectiveness. However, when both were applied together, their combined effect seemed to reduce compared to their individual applications. The scenario flipped in posterior brain regions, where individual applications of tDCS or tRNS underperformed compared to sham stimulation, but their combination shows improved efficacy. This pattern suggests that the observed effects might not stem directly from electrical stimulation but rather from an induced reorganization in functional connectivity, affecting even areas not directly stimulated.

**Figure 8:**
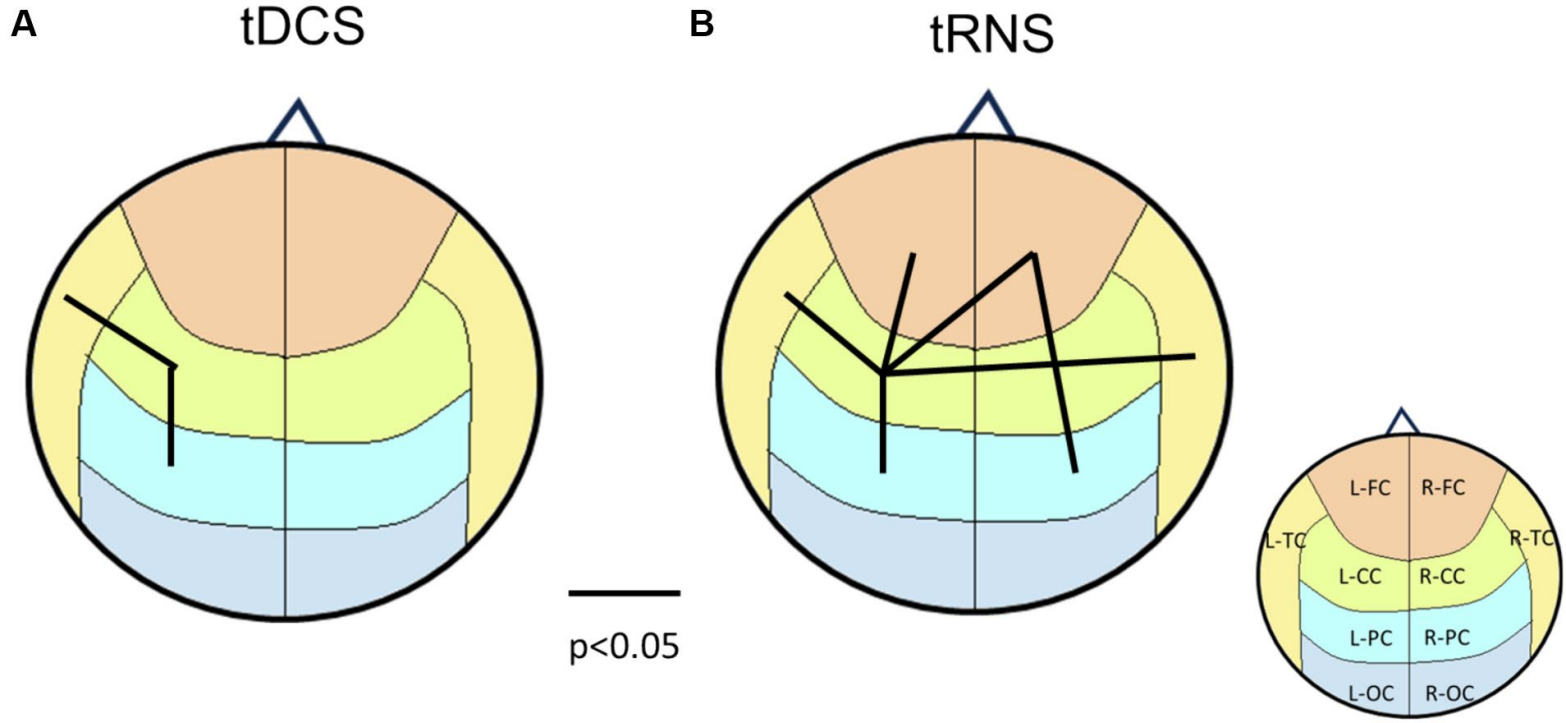
Brain connectivity map according to the p-values from paired-t tests between sham and tDCS/tRNS conditions. **(A)** The main effects of tDCS on brain connectivity, indicating left hemisphere lateralization. **(B)** The main effects of tRNS, revealing a more dispersed impact. The underlying brain regions are demarcated on the map, color-coded according to Figure 1C for identification.

When applying paired-t tests to (tDCS, sham) and (tRNS, sham) groups with *p* < 0.05 as the threshold for brain mapping, the results depicted in Figure 8 reveal pronounced lateralization effects induced by tDCS, which differ from those observed in the 2×2 statistical analysis in Figure 3. Notably, Figure 8 does not include data from the combined tDCS and tRNS stimulation, highlighting the discrepancy. This suggests that the complex, nonlinear interactions between tDCS and tRNS result in interactions that are not straightforwardly additive but rather interact in a more intricate and potentially compensatory manner.

Specifically, while tDCS typically enhances cortical excitability and induces lateralization towards the stimulated hemisphere, the addition of tRNS appears to modify this effect. tRNS, known for increasing neuronal noise and possibly enhancing dispersion across the cortex, may dilute or even reverse the directional excitatory effects of tDCS. This could result from the stochastic resonance introduced by tRNS, which competes with the focal excitatory influence of tDCS, leading to unexpected patterns of brain activation. Consequently, the lateralization effects observed with tDCS alone are not replicated when tRNS is added, suggesting a potentially antagonistic interaction between these neuromodulatory techniques.

The results from tRNS alone align with those from the 2 × 2 repeated measures ANOVA, indicating consistent effects across analyses when tRNS is applied independently. Further exploration through advanced modeling and a broader range of stimulation parameters is recommended to unravel the underlying mechanisms of these interactions.

Some studies for brain modeling assume that the brain responds in a monotonic and linear manner (Bikson et al., 2015). However, our study indicates that this assumption may require reevaluation. This perspective is supported by various dose–response studies (Giordano et al., 2017; Jamil et al., 2017), which also suggest the need for a revised understanding of brain response dynamics in the context of electrical stimulation. These studies collectively challenge the conventional linear response models and reveal the complexity of brain reactions to neuromodulatory interventions.

In relation to CSP results, we explored the combined influence of tDCS and tRNS on cortical inhibition. CSP is often less emphasized compared to cortical excitability typically assessed through Motor Evoked Potentials (MEP), but it is crucial due to the sensitivity of tDCS/tRNS-induced plasticity to GABAb receptors, with evidence suggesting prolonged stimulation can alter GABAb levels (Stagg et al., 2014; Bachtiar et al., 2015; Chaieb et al., 2015). The existing research on the impact of tDCS on CSP has shown mixed results; one study indicates anodal tDCS shortens CSP (Tremblay et al., 2013), whereas another finds no significant effect (Suzuki et al., 2012). Our study contributes to this discourse by demonstrating an increase in CSP with tDCS application. These discrepancies may arise from varying stimulation parameters and locations, highlighting the necessity for further investigations into the relationship between these parameters and CSP effects.

Comparing our findings with MEP results, prior studies have consistently demonstrated that both tDCS and tRNS enhance cortical excitability (Nitsche et al., 2008; Terney et al., 2008; Moliadze et al., 2010). However, when used in combination, these modalities yield more complex outcomes, sometimes contradicting the results observed with tRNS alone (Terney et al., 2008; Ho et al., 2015). This inconsistency suggests that the effects of these stimulations may vary based on different parameter combinations, and the precise underlying mechanisms require additional exploration (Bikson et al., 2018; Esmaeilpour et al., 2018; van der Groen et al., 2022). Notably, Bergmann et al. (2009) reported that the application of both tDCS and tDCS combined with transcranial alternating current stimulation (tACS) increases MEP amplitude during stimulation, indicating a possible overlap in mechanisms with tRNS.

The CBT test serves as an evaluative measure for visuospatial working memory, offering insights into the effects of electrical stimulation on working memory and response times. Our data indicate that tDCS enhances performance specifically in tasks requiring forward interference processing, whereas tRNS seems to improve reaction times in tasks with backward condition demands. These outcomes indicate a nuanced effectiveness of electrical stimulation in cognitive enhancement. The literature presents a spectrum of results, with certain studies reporting enhancements in learning performance (Fertonani et al., 2011; Brem et al., 2018) and working memory (Mulquiney et al., 2011; Holmes et al., 2016; Murphy et al., 2020) attributable to tDCS or tRNS. Our study corroborates these findings to some extent yet highlights the necessity for further investigation.

Additionally, Ke et al. (2024) suggest that the effectiveness of tRNS might be more pronounced under conditions of increased cognitive demand. This proposition is supported by our findings in the CBT, where tRNS notably enhanced reaction times specifically in the backward task condition, which poses a greater cognitive challenge compared to the forward task condition. This observation suggests the potential for tRNS effects to become more apparent when cognitive systems are actively engaged, aligning with the notion that neuromodulation outcomes may vary significantly with the cognitive load during stimulation. Further comparative analysis with the work of Murphy et al. (2020), who conducted electrical stimulation alongside a memory task, implies that the interaction of cognitive load and stimulation can significantly influence neuromodulatory effects (Murphy et al., 2020). Unlike their study, where stimulation and cognitive tasks were simultaneous, our approach measured cognitive performance separately from stimulation, possibly contributing to the observed differences in outcomes. This contrast highlights the potential for tRNS and tDCS to interact differently with cognitive processes depending on the presence or absence of concurrent cognitive demands. Thus, integrating a variety of neuroimaging techniques and cognitive assessments in future research could provide a more comprehensive understanding of how electrical stimulation affects the brain.

Furthermore, our observations from resting-state fMRI, CSP and CBT collectively indicate an intriguing non-synergistic interaction between tDCS and tRNS, potentially rooted in their different underlying mechanisms (Heitz and Schall, 2012). tDCS seems to enhance accuracy in attentional tasks by forging new neural pathways or reinforcing existing connections within the brain’s network (Coffman et al., 2012; Morales-Quezada et al., 2015; Miler et al., 2018). In contrast, tRNS appears to improve response speed by modulating neural synchronization through the mechanism of stochastic resonance – a process of adding subtle noise to enhance the brain’s non-linear system transmission (Schwarzkopf et al., 2011; Popescu et al., 2016; Ghin et al., 2018). However, their simultaneous application might lead to a decrease in overall efficacy, suggesting a complex interplay between their respective mechanisms (Lema et al., 2021).

Finally, the interaction effects of tDCS and tRNS on the temporal cortex reveal the significance of the choice of stimulation site, suggesting that the precise location of electrical stimulation may differentially influence cognitive functions. This implication necessitates additional research to ascertain the full scope of these neuromodulatory techniques and to refine their application for therapeutic purposes.

## 5 Limitations

**Figure 9:**
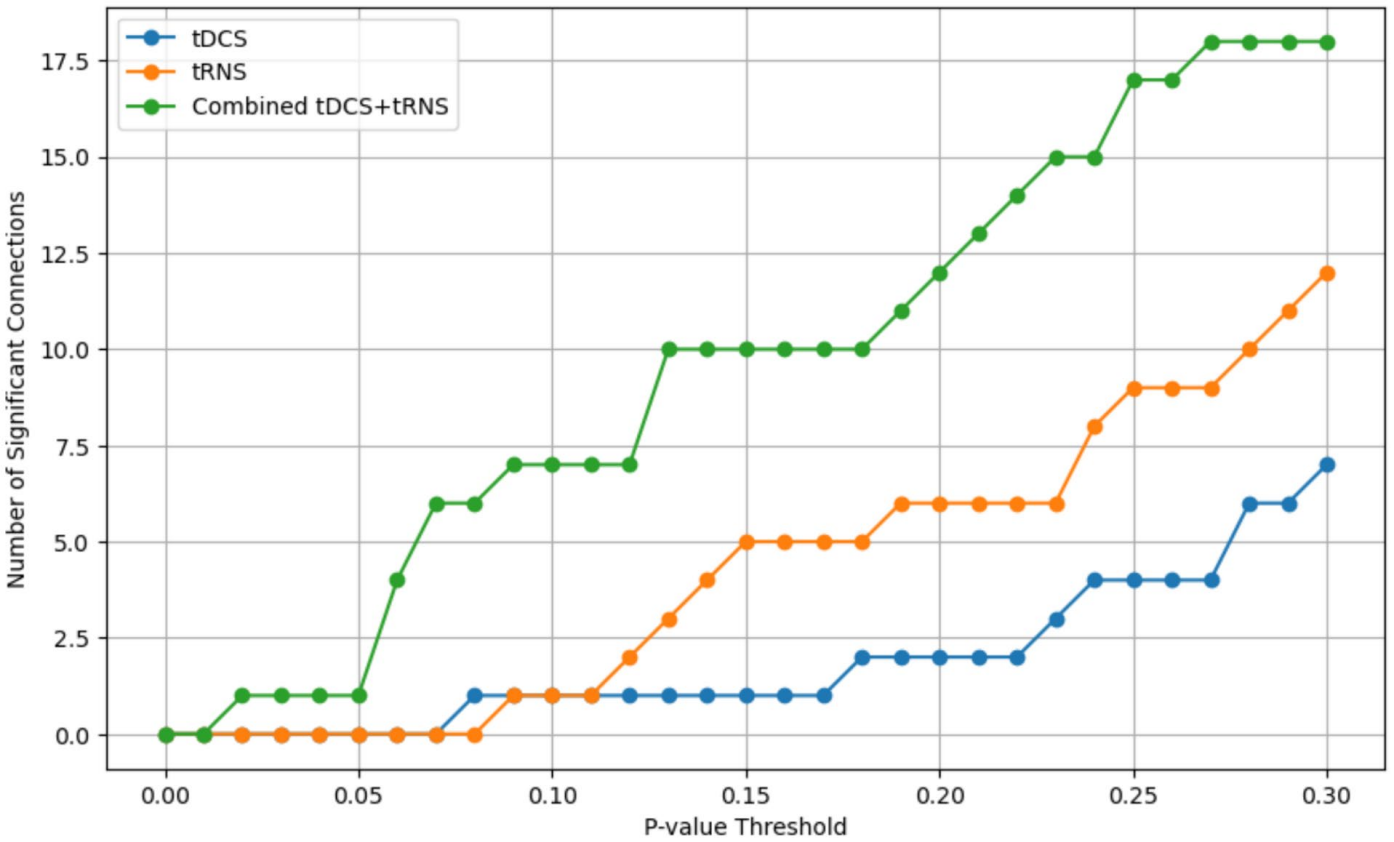
The number of statistically significant connectivity pairs identified at varying *p*-value thresholds, ranging from 0 to 0.3. The x-axis represents the *p*-value thresholds, and the y-axis indicates the count of significant connections. Separate lines are plotted for each stimulation condition: tDCS (blue), tRNS (yellow), and the combined tDCS+tRNS (green).

While this research offers valuable insights into the effects of tDCS and tRNS on the temporal cortex, there are several limitations. Firstly, while our study utilized a within-subject design to minimize inter-individual variability and enhance the reliability of our findings, the homogeneity of the participant group (all male students) and the small sample size might still impact the generalizability of the results. Additionally, our analysis confirmed that the connectivity results were robust across various *p*-value thresholds, reinforcing the consistency of our findings despite the sample size (Figure 9). Secondly, our focus solely on the temporal cortex using only resting-state functional connectivity may limit the applicability of our results to other brain areas and cognitive outcomes. Lastly, the initial exploration of the combined effects of tDCS and tRNS highlighted the need for more extensive, diverse research to fully understand their interactions. Future studies addressing these limitations are crucial for a comprehensive understanding of noninvasive brain stimulation techniques.

## 6 Conclusion

This study provides a comprehensive examination of the effects of tDCS and tRNS on the temporal cortex. Our observations across resting-state fMRI, CSP, and CBT indicate non-synergistic interactions between these two forms of brain stimulation, revealing the nuanced interplay of these two modalities. Overall, this study contributes to understanding the potential of tDCS and tRNS in cognitive enhancement and neurological disorder treatments, setting a foundation for future investigations in the field of noninvasive brain stimulation.

### Declaration of generative AI and AI-assisted technologies in the writing process

During the preparation of this work, the authors used ChatGPT to assist with language refinement and to enhance readability. After using this tool, the authors thoroughly reviewed and edited the content as needed. The authors take full responsibility for the content of the publication, ensuring its accuracy, integrity, and compliance with ethical standards.

## Data availability statement

The raw data supporting the conclusions of this article will be made available by the authors, without undue reservation.

## Ethics statement

The studies involving humans were approved by Institutional Review Board of National Cheng Kung Hospital, Tainan, Taiwan. The studies were conducted in accordance with the local legislation and institutional requirements. Written informed consent for participation in this study was provided by the participants' legal guardians/next of kin.

## Author contributions

P-JW: Conceptualization, Data curation, Formal analysis, Methodology, Software, Visualization, Writing – original draft, Writing – review & editing. C-HH: Data curation, Investigation, Software, Visualization, Writing – review & editing. S-YL: Resources, Writing – review & editing. AC: Conceptualization, Writing – review & editing. W-CW: Investigation, Writing – review & editing. C-CL: Funding acquisition, Methodology, Supervision, Writing – original draft, Writing – review & editing.

## References

[ref1] Bachtiar V. Near J. Johansen-Berg H. Stagg C. J. (2015). Modulation of GABA and resting state functional connectivity by transcranial direct current stimulation. eLife 4:e08789. doi: 10.7554/eLife.08789, PMID: 26381352 PMC4654253

[ref2] Badawy R. A. Vogrin S. J. Lai A. Cook M. J. (2013). The cortical excitability profile of temporal lobe epilepsy. Epilepsia 54, 1942–1949. doi: 10.1111/epi.12374, PMID: 24112043

[ref3] Baudewig J. Nitsche M. A. Paulus W. Frahm J. (2001). Regional modulation of BOLD MRI responses to human sensorimotor activation by transcranial direct current stimulation. Magn. Reson. Med. 45, 196–201. doi: 10.1002/1522-2594(200102)45:2<196::aid-mrm1026>3.0.co;2-1, PMID: 11180425

[ref4] Bergmann T. O. Groppa S. Seeger M. Molle M. Marshall L. Siebner H. R. (2009). Acute changes in motor cortical excitability during slow oscillatory and constant anodal transcranial direct current stimulation. J. Neurophysiol. 102, 2303–2311. doi: 10.1152/jn.00437.2009, PMID: 19692511

[ref5] Bestmann S. Walsh V. (2017). Transcranial electrical stimulation. Curr. Biol. 27, R1258–R1262. doi: 10.1016/j.cub.2017.11.001 29207262

[ref6] Bikson M. Brunoni A. R. Charvet L. E. Clark V. P. Cohen L. G. Deng Z. D.. (2018). Rigor and reproducibility in research with transcranial electrical stimulation: an NIMH-sponsored workshop. Brain Stimul. 11, 465–480. doi: 10.1016/j.brs.2017.12.008, PMID: 29398575 PMC5997279

[ref7] Bikson M. Truong D. Q. Mourdoukoutas A. P. Aboseria M. Khadka N. Adair D.. (2015). Modeling sequence and quasi-uniform assumption in computational neurostimulation. Prog. Brain Res. 222, 1–23. doi: 10.1016/bs.pbr.2015.08.005, PMID: 26541374

[ref8] Bouchard A. E. Renauld E. Fecteau S. (2023). Changes in resting-state functional MRI connectivity during and after transcranial direct current stimulation in healthy adults. Front. Hum. Neurosci. 17:1229618. doi: 10.3389/fnhum.2023.1229618, PMID: 37545594 PMC10398567

[ref9] Brem A. K. Almquist J. N. F. Mansfield K. Plessow F. Sella F. Santarnecchi E.. (2018). Modulating fluid intelligence performance through combined cognitive training and brain stimulation. Neuropsychologia 118, 107–114. doi: 10.1016/j.neuropsychologia.2018.04.008, PMID: 29649503

[ref10] Brunoni A. R. Sampaio-Junior B. Moffa A. H. Aparicio L. V. Gordon P. Klein I.. (2019). Noninvasive brain stimulation in psychiatric disorders: a primer. Braz. J. Psychiatry 41, 70–81. doi: 10.1590/1516-4446-2017-0018, PMID: 30328957 PMC6781710

[ref11] Chaieb L. Antal A. Paulus W. (2015). Transcranial random noise stimulation-induced plasticity is NMDA-receptor independent but sodium-channel blocker and benzodiazepines sensitive. Front. Neurosci. 9:125. doi: 10.3389/fnins.2015.00125 25914617 PMC4392589

[ref12] Coffman B. A. Trumbo M. C. Clark V. P. (2012). Enhancement of object detection with transcranial direct current stimulation is associated with increased attention. BMC Neurosci. 13, 1–8. doi: 10.1186/1471-2202-13-108 22963503 PMC3494452

[ref13] Dasilva A. F. Mendonca M. E. Zaghi S. Lopes M. Dossantos M. F. Spierings E. L.. (2012). tDCS-induced analgesia and electrical fields in pain-related neural networks in chronic migraine. Headache 52, 1283–1295. doi: 10.1111/j.1526-4610.2012.02141.x, PMID: 22512348 PMC4166674

[ref14] Esmaeilpour Z. Marangolo P. Hampstead B. M. Bestmann S. Galletta E. Knotkova H.. (2018). Incomplete evidence that increasing current intensity of tDCS boosts outcomes. Brain Stimul. 11, 310–321. doi: 10.1016/j.brs.2017.12.002, PMID: 29258808 PMC7050474

[ref15] Esmaeilpour Z. Shereen A. D. Ghobadi-Azbari P. Datta A. Woods A. J. Ironside M.. (2020). Methodology for tDCS integration with fMRI. Hum. Brain Mapp. 41, 1950–1967. doi: 10.1002/hbm.24908, PMID: 31872943 PMC7267907

[ref16] Fertonani A. Pirulli C. Miniussi C. (2011). Random noise stimulation improves neuroplasticity in perceptual learning. J. Neurosci. 31, 15416–15423. doi: 10.1523/JNEUROSCI.2002-11.2011, PMID: 22031888 PMC6703532

[ref17] Fregni F. Boggio P. S. Santos M. C. Lima M. Vieira A. L. Rigonatti S. P.. (2006). Noninvasive cortical stimulation with transcranial direct current stimulation in Parkinson's disease. Mov. Disord. 21, 1693–1702. doi: 10.1002/mds.21012 16817194

[ref18] Ghin F. Pavan A. Contillo A. Mather G. (2018). The effects of high-frequency transcranial random noise stimulation (hf-tRNS) on global motion processing: an equivalent noise approach. Brain Stimul. 11, 1263–1275. doi: 10.1016/j.brs.2018.07.048, PMID: 30078542

[ref19] Ghobadi-Azbari P. Jamil A. Yavari F. Esmaeilpour Z. Malmir N. Mahdavifar-Khayati R.. (2021). fMRI and transcranial electrical stimulation (tES): a systematic review of parameter space and outcomes. Prog. Neuro-Psychopharmacol. Biol. Psychiatry 107:110149. doi: 10.1016/j.pnpbp.2020.110149, PMID: 33096158

[ref20] Giordano J. Bikson M. Kappenman E. S. Clark V. P. Coslett H. B. Hamblin M. R.. (2017). Mechanisms and effects of transcranial direct current stimulation. Dose Response 15:1559325816685467. doi: 10.1177/1559325816685467, PMID: 28210202 PMC5302097

[ref21] Heitz R. P. Schall J. D. (2012). Neural mechanisms of speed-accuracy tradeoff. Neuron 76, 616–628. doi: 10.1016/j.neuron.2012.08.030, PMID: 23141072 PMC3576837

[ref22] Ho K. A. Taylor J. L. Loo C. K. (2015). Comparison of the effects of transcranial random noise stimulation and transcranial direct current stimulation on motor cortical excitability. J. ECT 31, 67–72. doi: 10.1097/YCT.0000000000000155 25010032

[ref23] Holmes J. Byrne E. M. Gathercole S. E. Ewbank M. P. (2016). Transcranial random noise stimulation does not enhance the effects of working memory training. J. Cogn. Neurosci. 28, 1471–1483. doi: 10.1162/jocn_a_00993 27315267

[ref24] Jamil A. Batsikadze G. Kuo H. I. Labruna L. Hasan A. Paulus W.. (2017). Systematic evaluation of the impact of stimulation intensity on neuroplastic after-effects induced by transcranial direct current stimulation. J. Physiol. 595, 1273–1288. doi: 10.1113/JP272738, PMID: 27723104 PMC5309387

[ref25] Julkunen P. Kallioniemi E. Kononen M. Saisanen L. (2013). Feasibility of automated analysis and inter-examiner variability of cortical silent period induced by transcranial magnetic stimulation. J. Neurosci. Methods 217, 75–81. doi: 10.1016/j.jneumeth.2013.04.019, PMID: 23660523

[ref26] Ke S.-C. Lo Y.-H. Tseng P. (2024). No frequency-specific effect of transcranial random noise stimulation on resting EEG. J. Integr. Neurosci. 23:59. doi: 10.31083/j.jin2303059, PMID: 38538231

[ref27] Kronberg G. Bridi M. Abel T. Bikson M. Parra L. C. (2017). Direct current stimulation modulates LTP and LTD: activity dependence and dendritic effects. Brain Stimul. 10, 51–58. doi: 10.1016/j.brs.2016.10.001, PMID: 28104085 PMC5260488

[ref28] Lee C. Jung Y. J. Lee S. J. Im C. H. (2017). COMETS2: an advanced MATLAB toolbox for the numerical analysis of electric fields generated by transcranial direct current stimulation. J. Neurosci. Methods 277, 56–62. doi: 10.1016/j.jneumeth.2016.12.008, PMID: 27989592

[ref29] Lema A. Carvalho S. Fregni F. Goncalves O. F. Leite J. (2021). The effects of direct current stimulation and random noise stimulation on attention networks. Sci. Rep. 11:6201. doi: 10.1038/s41598-021-85749-7, PMID: 33737661 PMC7973424

[ref30] Li L. M. Violante I. R. Leech R. Ross E. Hampshire A. Opitz A.. (2019). Brain state and polarity dependent modulation of brain networks by transcranial direct current stimulation. Hum. Brain Mapp. 40, 904–915. doi: 10.1002/hbm.24420, PMID: 30378206 PMC6387619

[ref31] Liebetanz D. Klinker F. Hering D. Koch R. Nitsche M. A. Potschka H.. (2006). Anticonvulsant effects of transcranial direct-current stimulation (tDCS) in the rat cortical ramp model of focal epilepsy. Epilepsia 47, 1216–1224. doi: 10.1111/j.1528-1167.2006.00539.x, PMID: 16886986

[ref32] Marquez-Ruiz J. Leal-Campanario R. Sanchez-Campusano R. Molaee-Ardekani B. Wendling F. Miranda P. C.. (2012). Transcranial direct-current stimulation modulates synaptic mechanisms involved in associative learning in behaving rabbits. Proc. Natl. Acad. Sci. USA 109, 6710–6715. doi: 10.1073/pnas.1121147109, PMID: 22493252 PMC3340065

[ref33] Marshall L. Molle M. Siebner H. R. Born J. (2005). Bifrontal transcranial direct current stimulation slows reaction time in a working memory task. BMC Neurosci. 6:23. doi: 10.1186/1471-2202-6-23, PMID: 15819988 PMC1090588

[ref34] Miler J. A. Meron D. Baldwin D. S. Garner M. (2018). The effect of prefrontal transcranial direct current stimulation on attention network function in healthy volunteers. Neuromodulation 21, 355–361. doi: 10.1111/ner.12629, PMID: 28714563

[ref35] Moliadze V. Antal A. Paulus W. (2010). Electrode-distance dependent after-effects of transcranial direct and random noise stimulation with extracephalic reference electrodes. Clin. Neurophysiol. 121, 2165–2171. doi: 10.1016/j.clinph.2010.04.033, PMID: 20554472

[ref36] Mondino M. Ghumman S. Gane C. Renauld E. Whittingstall K. Fecteau S. (2019). Effects of transcranial stimulation with direct and alternating current on resting-state functional connectivity: an exploratory study simultaneously combining stimulation and multiband functional magnetic resonance imaging. Front. Hum. Neurosci. 13:474. doi: 10.3389/fnhum.2019.00474, PMID: 32116597 PMC7012783

[ref37] Morales-Quezada L. Cosmo C. Carvalho S. Leite J. Castillo-Saavedra L. Rozisky J. R.. (2015). Cognitive effects and autonomic responses to transcranial pulsed current stimulation. Exp. Brain Res. 233, 701–709. doi: 10.1007/s00221-014-4147-y, PMID: 25479736

[ref38] Mulquiney P. G. Hoy K. E. Daskalakis Z. J. Fitzgerald P. B. (2011). Improving working memory: exploring the effect of transcranial random noise stimulation and transcranial direct current stimulation on the dorsolateral prefrontal cortex. Clin. Neurophysiol. 122, 2384–2389. doi: 10.1016/j.clinph.2011.05.009 21665534

[ref39] Murphy O. W. Hoy K. E. Wong D. Bailey N. W. Fitzgerald P. B. Segrave R. A. (2020). Transcranial random noise stimulation is more effective than transcranial direct current stimulation for enhancing working memory in healthy individuals: Behavioural and electrophysiological evidence. Brain Stimul. 13, 1370–1380. doi: 10.1016/j.brs.2020.07.001, PMID: 32659482

[ref40] Nitsche M. A. Cohen L. G. Wassermann E. M. Priori A. Lang N. Antal A.. (2008). Transcranial direct current stimulation: state of the art 2008. Brain Stimul. 1, 206–223. doi: 10.1016/j.brs.2008.06.004, PMID: 20633386

[ref41] Nitsche M. A. Seeber A. Frommann K. Klein C. C. Rochford C. Nitsche M. S.. (2005). Modulating parameters of excitability during and after transcranial direct current stimulation of the human motor cortex. J. Physiol. 568, 291–303. doi: 10.1113/jphysiol.2005.092429, PMID: 16002441 PMC1474757

[ref42] Peña J. Muthalib M. Sampedro A. Cardoso-Botelho M. Zabala O. Ibarretxe-Bilbao N.. (2023). Enhancing creativity with combined transcranial direct current and random noise stimulation of the left dorsolateral prefrontal cortex and inferior frontal gyrus. J. Creat. Behav. 57, 65–81. doi: 10.1002/jocb.562

[ref43] Popescu T. Krause B. Terhune D. B. Twose O. Page T. Humphreys G.. (2016). Transcranial random noise stimulation mitigates increased difficulty in an arithmetic learning task. Neuropsychologia 81, 255–264. doi: 10.1016/j.neuropsychologia.2015.12.028, PMID: 26731199 PMC4749538

[ref44] Radman T. Ramos R. L. Brumberg J. C. Bikson M. (2009). Role of cortical cell type and morphology in subthreshold and suprathreshold uniform electric field stimulation in vitro. Brain Stimul. 2, 215–228.e3. doi: 10.1016/j.brs.2009.03.007, PMID: 20161507 PMC2797131

[ref45] Schwarzkopf D. S. Silvanto J. Rees G. (2011). Stochastic resonance effects reveal the neural mechanisms of transcranial magnetic stimulation. J. Neurosci. 31, 3143–3147. doi: 10.1523/JNEUROSCI.4863-10.2011, PMID: 21368025 PMC3059801

[ref46] Stagg C. J. Antal A. Nitsche M. A. (2018). Physiology of transcranial direct current stimulation. J. ECT 34, 144–152. doi: 10.1097/YCT.0000000000000510 29877965

[ref47] Stagg C. J. Bachtiar V. Amadi U. Gudberg C. A. Ilie A. S. Sampaio-Baptista C.. (2014). Local GABA concentration is related to network-level resting functional connectivity. eLife 3:e01465. doi: 10.7554/eLife.01465, PMID: 24668166 PMC3964822

[ref48] Stagg C. J. O'Shea J. Kincses Z. T. Woolrich M. Matthews P. M. Johansen-Berg H. (2009). Modulation of movement-associated cortical activation by transcranial direct current stimulation. Eur. J. Neurosci. 30, 1412–1423. doi: 10.1111/j.1460-9568.2009.06937.x, PMID: 19788568

[ref49] Suzuki K. Fujiwara T. Tanaka N. Tsuji T. Masakado Y. Hase K.. (2012). Comparison of the after-effects of transcranial direct current stimulation over the motor cortex in patients with stroke and healthy volunteers. Int. J. Neurosci. 122, 675–681. doi: 10.3109/00207454.2012.707715, PMID: 22747238

[ref50] Terney D. Chaieb L. Moliadze V. Antal A. Paulus W. (2008). Increasing human brain excitability by transcranial high-frequency random noise stimulation. J. Neurosci. 28, 14147–14155. doi: 10.1523/JNEUROSCI.4248-08.2008, PMID: 19109497 PMC6671476

[ref51] Tremblay S. Beaule V. Lepage J. F. Theoret H. (2013). Anodal transcranial direct current stimulation modulates GABAB-related intracortical inhibition in the M1 of healthy individuals. Neuroreport 24, 46–50. doi: 10.1097/WNR.0b013e32835c36b8, PMID: 23196416

[ref52] van der Groen O. Potok W. Wenderoth N. Edwards G. Mattingley J. B. Edwards D. (2022). Using noise for the better: the effects of transcranial random noise stimulation on the brain and behavior. Neurosci. Biobehav. Rev. 138:104702. doi: 10.1016/j.neubiorev.2022.104702, PMID: 35595071

[ref53] van der Groen O. Wenderoth N. (2016). Transcranial random noise stimulation of visual cortex: stochastic resonance enhances central mechanisms of perception. J. Neurosci. 36, 5289–5298. doi: 10.1523/JNEUROSCI.4519-15.2016, PMID: 27170126 PMC6601807

[ref54] Whitfield-Gabrieli S. Nieto-Castanon A. (2012). Conn: a functional connectivity toolbox for correlated and anticorrelated brain networks. Brain Connect. 2, 125–141. doi: 10.1089/brain.2012.0073, PMID: 22642651

