# Supplementary material for: The distinct and potentially conflicting effects of tDCS and tRNS on brain connectivity, cortical inhibition, and visuospatial memory
Source: Front Hum Neurosci. 2024 May 30;18:1415904. doi: 10.3389/fnhum.2024.1415904 (PMC11169625; doi:10.3389/fnhum.2024.1415904)
Supplement: Supplementary file 1 [file Table_1.docx]

Supplementary Material

# Supplementary Figures and Tables

## Supplementary Table

**Table S1.** List of cortical areas and the corresponding Harvard-Oxford atlas.

| **Cortical area** | **Harvard-Oxford atlas** | **Cortical area** | **Harvard-Oxford atlas** |
| --- | --- | --- | --- |
| Left frontal cortex  (L-FC) | atlas.FP l (Frontal Pole Left)  atlas.SFG l (Superior Frontal Gyrus Left)  atlas.MidFG l (Middle Frontal Gyrus Left)  atlas.IFG tri l (Inferior Frontal Gyrus, pars triangularis Left)  atlas.IFG oper l (Inferior Frontal Gyrus, pars opercularis Left)  atlas.SMA L Juxtapositional Lobule Cortex -formerly Supplementary Motor Cortex- Left)  atlas.FOrb l (Frontal Orbital Cortex Left)  atlas.FO l (Frontal Operculum Cortex Left) | Right frontal cortex  (R-FC) | atlas.FP r (Frontal Pole Right)  atlas.SFG r (Superior Frontal Gyrus Right)  atlas.MidFG r (Middle Frontal Gyrus Right)  atlas.IFG tri r (Inferior Frontal Gyrus, pars triangularis Right)  atlas.IFG oper r (Inferior Frontal Gyrus, pars opercularis Right)  atlas.SMA r (Juxtapositional Lobule Cortex -formerly Supplementary Motor Cortex- Right)  atlas.FOrb r (Frontal Orbital Cortex Right)  atlas.FO r (Frontal Operculum Cortex Right) |
| Left central cortex  (L-CC) | atlas.PreCG l (Precentral Gyrus Left)  atlas.PostCG l (Postcentral Gyrus Left)  atlas.CO l (Central Opercular Cortex Left)  atlas.CO l (Central Opercular Cortex Left) | Right central cortex  (R-CC) | atlas.PreCG r (Precentral Gyrus Right)  atlas.PostCG r (Postcentral Gyrus Right)  atlas.CO r (Central Opercular Cortex Right) |
| Left temporal cortex  (L-TC) | atlas.TP l (Temporal Pole Left)  atlas.aSTG l (Superior Temporal Gyrus, anterior division Left)  atlas.pSTG l (Superior Temporal Gyrus, posterior division Left)  atlas.aMTG l (Middle Temporal Gyrus, anterior division Left)  atlas.pMTG l (Middle Temporal Gyrus, posterior division Left)  atlas.toMTG l (Middle Temporal Gyrus, temporooccipital part Left)  atlas.aITG l (Inferior Temporal Gyrus, anterior division Left)  atlas.pITG l (Inferior Temporal Gyrus, posterior division Left)  atlas.toITG l (Inferior Temporal Gyrus, temporooccipital part Left)  atlas.aTFusC l (Temporal Fusiform Cortex, anterior division Left)  atlas.pTFusC l (Temporal Fusiform Cortex, posterior division Left)  atlas.TOFusC l (Temporal Occipital Fusiform Cortex Left)  atlas.PP l (Planum Polare Left)  atlas.HG l (Heschl's Gyrus Left)  atlas.PT l (Planum Temporale Left) | Right temporal cortex  (R-TC) | atlas.TP r (Temporal Pole Right)  atlas.aSTG r (Superior Temporal Gyrus, anterior division Right)  atlas.pSTG r (Superior Temporal Gyrus, posterior division Right)  atlas.aMTG r (Middle Temporal Gyrus, anterior division Right)  atlas.pMTG r (Middle Temporal Gyrus, posterior division Right)  atlas.toMTG r (Middle Temporal Gyrus, temporooccipital part Right)  atlas.aITG r (Inferior Temporal Gyrus, anterior division Right)  atlas.pITG r (Inferior Temporal Gyrus, posterior division Right)  atlas.toITG r (Inferior Temporal Gyrus, temporooccipital part Right)  atlas.aTFusC r (Temporal Fusiform Cortex, anterior division Right)  atlas.pTFusC r (Temporal Fusiform Cortex, posterior division Right)  atlas.TOFusC r (Temporal Occipital Fusiform Cortex Right)  atlas.PP r (Planum Polare Right)  atlas.HG r (Heschl's Gyrus Right)  atlas.PT r (Planum Temporale Right) |
| Left parietal cortex  (L-PC) | atlas.SPL l (Superior Parietal Lobule Left)  atlas.aSMG l (Supramarginal Gyrus, anterior division Left)  atlas.pSMG l (Supramarginal Gyrus, posterior division Left)  atlas.AG l (Angular Gyrus Left)  atlas.PO l (Parietal Operculum Cortex Left) | Right parietal cortex  (R-PC) | atlas.SPL r (Superior Parietal Lobule Right)  atlas.aSMG r (Supramarginal Gyrus, anterior division Right)  atlas.pSMG r (Supramarginal Gyrus, posterior division Right)  atlas.AG r (Angular Gyrus Right)  atlas.PO r (Parietal Operculum Cortex Right) |
| Left occipital cortex  (L-OC) | atlas.sLOC l (Lateral Occipital Cortex, superior division Left)  atlas.iLOC l (Lateral Occipital Cortex, inferior division Left)  atlas.ICC l (Intracalcarine Cortex Left)  atlas.Cuneal l (Cuneal Cortex Left)  atlas.OFusG l (Occipital Fusiform Gyrus Left)  atlas.SCC l (Supracalcarine Cortex Left)  atlas.OP l (Occipital Pole Left) | Right occipital cortex  (R-OC) | atlas.sLOC r (Lateral Occipital Cortex, superior division Right)  atlas.iLOC r (Lateral Occipital Cortex, inferior division Right)  atlas.ICC r (Intracalcarine Cortex Right)  atlas.Cuneal r (Cuneal Cortex Right)  atlas.OFusG r (Occipital Fusiform Gyrus Right)  atlas.SCC r (Supracalcarine Cortex Right)  atlas.OP r (Occipital Pole Right) |
